# Comparative associations of immune-inflammatory, immune-lipid, and metabolic composite indices with hyperuricemia among Chinese adults with glycemic abnormalities: a health examination-based cross-sectional study

**DOI:** 10.3389/fendo.2026.1927643

**Published:** 2026-09-14

**Authors:** Xinru Sun, Jun Zhou, Ling Zhou, Yuhong Zheng, Jiayi Ren, Zhifeng Jiang, Xinmiao Wang, Jiaqi Zhang, Zeyin Xin, Yi Kong, Bin Li, Yizhan Wang

**Affiliations:** 1 Center of Acupuncture and Moxibustion, Beijing Hospital of Traditional Chinese Medicine, Capital Medical University, Beijing, China; 2 Beijing Key Laboratory of Digital and Intelligent Acupuncture, Beijing Hospital of Traditional Chinese Medicine, Capital Medical University, Beijing, China; 3 School of Printing and Packaging Engineering, Beijing Institute of Graphic Communication, Beijing, China; 4 Preventive Treatment Center, Beijing Hospital of Traditional Chinese Medicine, Capital Medical University, Beijing, China; 5 Graduate School, Beijing University of Chinese Medicine, Beijing, China; 6 Department of General Internal Medicine, Eye Hospital, China Academy of Chinese Medical Sciences, Beijing, China

**Keywords:** glycemic abnormalities, hyperuricemia, immune-inflammatory indices, immune-lipid indices, metabolic composite indices

## Abstract

**Background:**

Hyperuricemia (HUA) commonly coexists with glycemic abnormalities and may reflect complex metabolic disturbances involving inflammation, lipid dysregulation, insulin resistance, and impaired renal urate handling. However, evidence directly comparing immune-inflammatory, immune–lipid, and metabolic composite indices in relation to HUA among individuals with glycemic abnormalities remains limited. This study aimed to compare the associations of these composite indices with HUA among Chinese adults with glycemic abnormalities.

**Methods:**

This large-sample health examination-based cross-sectional study included 10, 089 Chinese adults with glycemic abnormalities, of whom 2, 735 had HUA. Immune-inflammatory indices, immune–lipid indices, and metabolic composite indices were calculated using routine laboratory and anthropometric parameters. Multi variable logistic regression models were used to evaluate the associations between each standardized composite index and HUA. Restricted cubic spline analyses were performed to explore nonlinear associations, and subgroup analyses were conducted according to sex, age, BMI, hypertension, fatty liver disease, smoking status, alcohol drinking, glycemic status and eGFR.

**Results:**

Among participants with glycemic abnormalities, those with HUA showed more pronounced immune–lipid and metabolic abnormalities than those without HUA. In primary multivariable-adjusted model, immune–lipid indices, including WHR, NHR, LHR, MHR, and PHR, were all positively associated with HUA. Among metabolic indices, AIP showed the strongest association with HUA, followed by TyG, METS-IR, and CHG, whereas RC and NHHR were not significantly associated with HUA in the primary multivariable-adjusted model. Among traditional immune-inflammatory indices, SIRI and PIV remained positively associated with HUA, while PLR showed an inverse association and NLR, MLR, and SII were not significant. Restricted cubic spline analyses revealed nonlinear and threshold-dependent associations for several immune–lipid and metabolic indices, with HUA odds increasing at lower-to-moderate levels and then tending to plateau or attenuate at higher levels. Among demographic and anthropometric characteristics, sex, age, and BMI were the most prominent effect modifiers, with generally stronger associations observed in women, participants aged <60 years, and those with BMI <24 kg/m².

**Conclusions:**

Among Chinese adults with glycemic abnormalities, all three categories of indices were associated with HUA. Among the metabolic composite indices, AIP showed the strongest association with HUA, while TyG, METS-IR, and CHG were also positively associated with HUA. Immune–lipid indices, including WHR, NHR, LHR, MHR, and PHR, showed consistent positive associations with HUA, whereas among conventional immune-inflammatory indicators, PLR, PIV, and SIRI were associated with HUA.

## 1 Introduction

Prediabetes and diabetes are common disorders of glucose metabolism and represent a major global public health challenge (1). Rather than representing hyperglycemia alone, glycemic abnormalities often occur in the context of broader metabolic perturbations, underscoring its role as a marker of systemic metabolic dysregulation (2, 3). Hyperuricemia (HUA) is one such metabolic disturbance that commonly coexists with glycemic abnormalities. Glycemic abnormalities have been linked to increased risks of all-cause and cardiovascular mortality (4, 5), and HUA has also been associated with similar adverse outcomes, especially among individuals with diabetes, cardiovascular disease, or kidney disease (6, 7). Elevated serum uric acid (SUA) has been associated with insulin resistance, dyslipidemia, impaired renal urate excretion, oxidative stress, endothelial dysfunction, and chronic inflammation, while insulin resistance may further promote urate retention by reducing renal uric acid clearance (8–10). Among individuals with abnormal glucose metabolism, HUA may represent more than a concomitant feature of metabolic dysregulation; rather, it may reflect the cumulative coexistence of insulin resistance, atherogenic lipid abnormalities, impaired renal urate handling, and vascular inflammatory burden (11, 12).

Given the involvement of inflammation, lipid metabolic abnormalities, and insulin resistance in the glycemic abnormalities–HUA relationship, readily available composite indices may help characterize these interconnected pathophysiological processes (13). Several composite biomarkers derived from routine laboratory parameters have been proposed to capture these interrelated processes. Immune-inflammatory indices, particularly the neutrophil-to-lymphocyte ratio (NLR) and systemic immune-inflammation index (SII), have been widely applied in studies of inflammation-related, metabolic, and cardiovascular diseases (14). High-density lipoprotein cholesterol (HDL-C)-related inflammatory–lipid indices, particularly the monocyte-to-HDL-C ratio (MHR) and neutrophil-to-HDL-C ratio (NHR), have been increasingly investigated as accessible markers integrating inflammatory and lipid metabolic information (15). MHR reflects the balance between monocyte-mediated inflammation and the anti-inflammatory, anti-atherogenic properties of HDL-C (16). NHR has recently been linked to cardiometabolic and vascular outcomes in populations with type 2 diabetes or cardiovascular disease (17). Metabolic indices, including the triglyceride-glucose (TyG) index, metabolic score for insulin resistance (METS-IR), atherogenic index of plasma (AIP), have also been reported to reflect insulin resistance, atherogenic lipid profiles, and cardiometabolic risk (18–20). Recent studies have further linked these composite indices to prediabetes, type 2 diabetes mellitus, metabolic syndrome, cardiovascular outcomes, and hyperuricemia.

Although composite indices may better reflect metabolic or cardiovascular risk than their individual components, most studies have focused on a single index or a limited set of indicators. Direct evidence comparing immune-inflammatory, immune–lipid, and metabolic composite indices in relation to hyperuricemia remains scarce, particularly among individuals with glycemic abnormalities. Therefore, comparing these indices within the same population may help clarify whether HUA is more closely related to inflammation, lipid metabolic abnormalities, or insulin resistance-related metabolic dysfunction.

## 2 Materials and methods

### 2.1 Study design

This retrospective cross-sectional study was conducted at Beijing Hospital of Traditional Chinese Medicine, Capital Medical University, and was jointly performed by the Preventive Treatment Center and Center of Acupuncture and Moxibustion. The results are reported in accordance with the STROBE (Strengthening the Reporting of Observational Studies in Epidemiology) statement for cross-sectional studies (https://www.strobe-statement.org/), and the checklist is provided in **Supplementary Table 1**.

### 2.2 Data sources

We retrospectively extracted data from adults who underwent routine health examinations at Beijing Hospital of Traditional Chinese Medicine, Capital Medical University, from January 2019 to December 2024. The extracted variables covered sociodemographic information, lifestyle-related characteristics, anthropometric and clinical examination findings, abdominal ultrasonography results, and laboratory measurements. Laboratory data included hematological parameters, glucose-related indices, lipid profiles, and markers of liver and kidney function. All assessments were performed according to uniform procedures throughout the study population.

Glycemic abnormalities were defined according to the diagnostic criteria of the American Diabetes Association (ADA) and the World Health Organization (WHO), including any of the following: fasting plasma glucose (FPG) ≥ 5.6 mmol/L (100 mg/dL), glycated hemoglobin (HbA1c) ≥ 5.7%, a previous diagnosis of diabetes mellitus, or current use of glucose-lowering medications (21, 22). HUA was defined as the SUA level ≥ 420 µmol/L (7 mg/dL) in men and ≥ 360 µmol/L (6 mg/dL) in women (23).

### 2.3 Calculation of composite indices

Based on routine hematological, lipid, glucose, and anthropometric parameters, a series of composite indices were calculated for subsequent analyses. The definitions and calculation formulas of these indices, including NLR, MLR, PLR, SIRI, SII, PIV, WHR, NHR, LHR, MHR, PHR, RC, NHHR, AIP, TyG, METS-IR, and CHG (24–28), are summarized in **Table 1**.

**Table 1:** Definitions and calculation formulas of composite inflammatory, lipid-related, and metabolic indices.

Index	Full name	Formula
Immune-inflammatory indices		
NLR	Neutrophil-to-lymphocyte ratio	NEUT/LYM
MLR	Monocyte-to-lymphocyte ratio	MONO/LYM
PLR	Platelet-to-lymphocyte ratio	PLT/LYM
SIRI	Systemic inflammatory response index	NEUT × MONO/LYM
SII	Systemic immune-inflammation index	PLT × NEUT/LYM
PIV	Pan-immune-inflammation value	NEUT × PLT × MONO/LYM
Immune-lipid indices		
WHR	White blood cell count-to-HDL-C ratio	WBC/HDL-C
NHR	Neutrophil-to-HDL-C ratio	NEUT/HDL-C
LHR	Lymphocyte-to-HDL-C ratio	LYM/HDL-C
MHR	Monocyte-to-HDL-C ratio	MONO/HDL-C
PHR	Platelet-to-HDL-C ratio	PLT/HDL-C
Metabolic composite indices		
RC	Remnant cholesterol	TC − LDL-C − HDL-C
NHHR	Non-HDL-C-to-HDL-C ratio	(TC − HDL-C)/HDL-C
AIP	Atherogenic index of plasma	log_10_(TG/HDL-C)
TyG	Triglyceride-glucose index	ln[(TG × FPG)/2]
METS-IR	Metabolic score for insulin resistance	{ln[2 × FPG + TG] × BMI}/ln(HDL-C)
CHG	Cholesterol, HDL-C, and glucose index	ln{[TC × FPG]/[2 × HDL-C]}

WBC, white blood cell count; NEUT, neutrophil count; LYM, lymphocyte count; MONO, monocyte count; PLT, platelet count; TC, total cholesterol; TG, triglycerides; HDL-C, high-density lipoprotein cholesterol; LDL-C, low-density lipoprotein cholesterol; FPG, fasting plasma glucose; BMI, body mass index.

### 2.4 Participants

Health examination participants were included if they met the following criteria: (1) age ≥18 years; (2) complete FPG and HbA1c data for identifying glycemic abnormalities; (3) complete SUA data for classifying HUA groups. (4) complete data on key covariates including hematological parameters, total cholesterol (TC), triglycerides (TG), high-density lipoprotein cholesterol (HDL-C), low-density lipoprotein cholesterol (LDL-C), body mass index (BMI), fatty liver status, and basic demographic characteristics. The exclusion criteria were as follows: (1) type 1 diabetes mellitus or secondary diabetes; (2) pregnancy or lactation; (3) acute inflammatory or infectious diseases at the time of data collection; (4) a documented diagnosis of severe cognitive impairment in the medical records; and (5) severe cardiovascular or cerebrovascular diseases or immune system diseases.

### 2.5 Sample size

This was a cross-sectional study. Multivariable logistic regression models were primarily used to examine the associations between composite indices related to immune inflammation and glucolipid metabolism and comorbidity status. The sample size estimation was based on the commonly used events per variable (EPV) principle for logistic regression analysis. To ensure the robustness of the models, an EPV criterion of 20 was adopted in this study (29). The required number of positive outcome events was calculated according to the 17 composite indices and 7 potential confounding variables, including age, sex, BMI, smoking history, alcohol drinking, hypertension, and fatty liver disease. Based on an EPV criterion of 20, at least 480 positive outcome events were required. The actual number of observed positive outcome events in this study exceeded the required number, indicating that the sample size was sufficient for subsequent analyses.

### 2.6 Statistical methods

HUA was treated as a binary outcome variable, with non-HUA serving as the reference group. The distributional characteristics of continuous variables were assessed within each group. Given the relatively large sample size, normality was evaluated based on the Shapiro–Wilk test, together with visual inspection of Q–Q plots and assessment of skewness, kurtosis, and outliers, to determine whether variables could be reasonably treated as approximately normally distributed. Continuous variables with an approximately normal distribution were presented as the mean ± standard deviation (SD) and compared between groups using the independent-samples t test; when the assumption of homogeneity of variance was not met, Welch’s t test was applied. Non-normally distributed variables were expressed as the median and interquartile range (IQR) and compared using the Mann–Whitney U test. Categorical variables were summarized as frequencies and percentages [n (%)] and compared between groups using Pearson’s χ² test. All continuous composite indicators were standardized using Z-score transformation before logistic regression analyses, whereas age and BMI were retained in their original units as adjustment covariates. Logistic regression models were constructed to examine the associations between each indicator and HUA. Model 1 was unadjusted; Model 2 was adjusted for age and sex; and Model 3 was further adjusted for age, sex, BMI, smoking history, alcohol drinking, hypertension, and fatty liver disease. Model 4 was additionally adjusted for glycemic status and estimated glomerular filtration rate (eGFR). Each indicator was entered into the logistic regression model separately. The associations between each indicator and HUA were reported as odds ratios (ORs) with 95% confidence intervals (CIs). Indicators that remained statistically significant in Model 3 were further examined using restricted cubic spline (RCS) analysis to assess potential nonlinear associations with HUA. The RCS models were adjusted for the same covariates as Model 3. The coding scheme for variables included in the logistic regression models is shown in **Supplementary Table 2**. The nonlinearity was assessed by comparing the model containing only the linear term with the model containing both linear and spline terms. Subgroup analyses were conducted across predefined subgroups of age, sex, BMI, hypertension, fatty liver disease, smoking status, alcohol drinking, glycemic status, and eGFR. Within each subgroup, multivariable logistic regression models were fitted to estimate ORs and 95% CIs for HUA per 1-SD increase in each Z-score–standardized composite index, with adjustment for age, sex, BMI, hypertension, fatty liver disease, smoking history, alcohol drinking, glycemic status, and eGFR, except for the corresponding stratification variable. Effect modification was assessed by adding multiplicative interaction terms to the fully adjusted model in the overall population. Interaction *P* values were adjusted using the Benjamini–Hochberg false discovery rate (FDR) method, and an FDR-adjusted *P* value < 0.05 was considered statistically significant.

## 3 Results

### 3.1 General information

A total of 19783 participants were initially screened. Among them, 8057 participants with normal glycemic status were excluded based on FPG and HbA1c data. Subsequently, 823 participants were excluded because of missing data on SUA, hematological parameters, lipid profiles, BMI, fatty liver disease status, or basic demographic characteristics. Of the remaining participants, 814 were further excluded because of type 1 diabetes mellitus or secondary diabetes, pregnancy or lactation, acute inflammatory or infectious diseases, severe cognitive impairment, severe cardiovascular or cerebrovascular diseases, or immune system diseases. Finally, 10089 participants were included in the final analysis. Details are shown in **Figure 1**.

**Figure 1:**
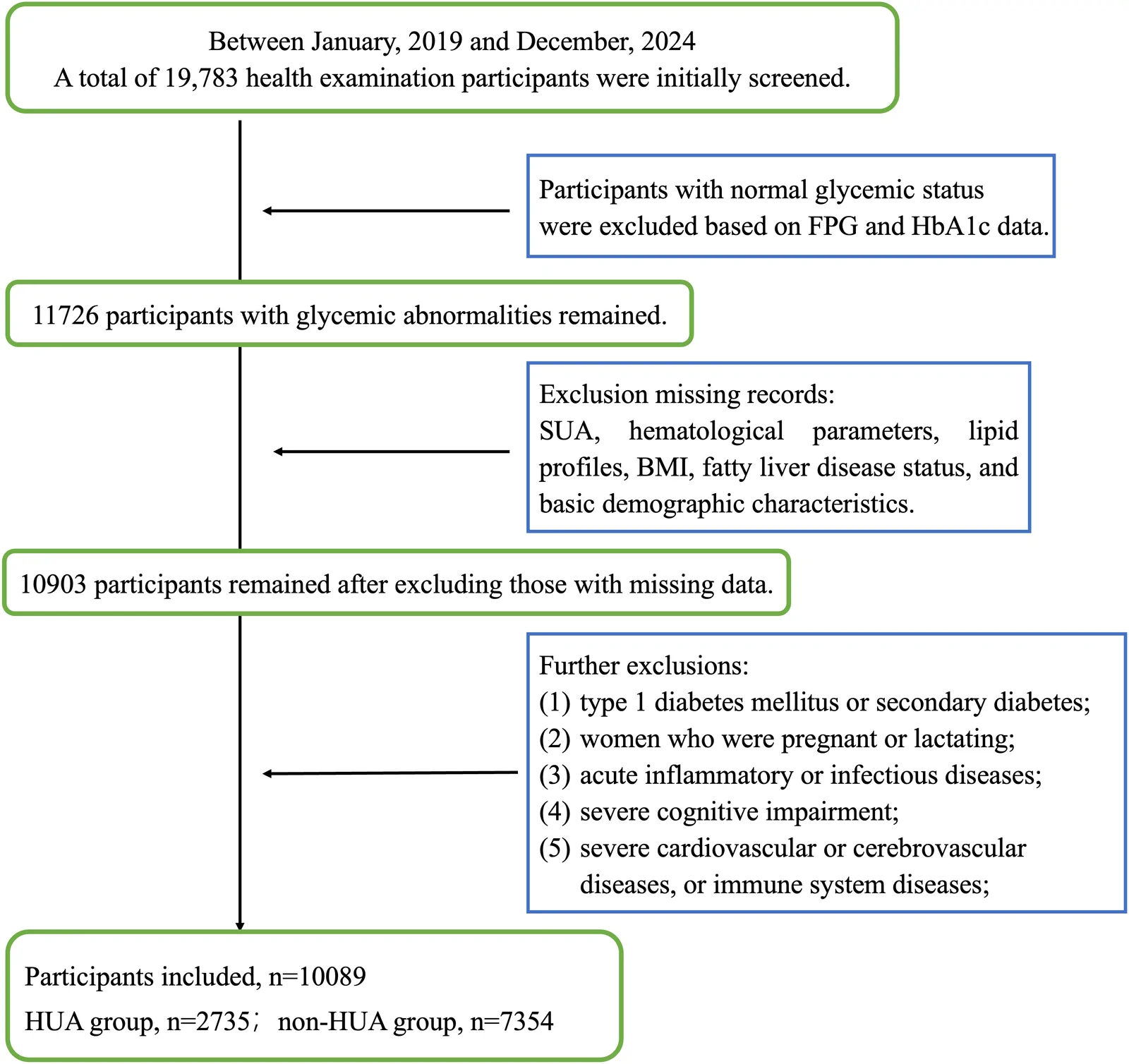
Flowchart of participant selection.

### 3.2 Baseline characteristics of the participants

Participants with glycemic abnormalities were categorized into the HUA group and the non-HUA group according to serum uric acid (SUA) levels. The baseline characteristics are presented in **Table 2**. A total of 10, 089 participants were included in this cross-sectional analysis, with a mean age of 59.22 ± 13.07 years; 45.5% were men and 54.5% were women. Among them, 2, 735 participants were classified as having HUA, whereas 7, 354 were classified as non-HUA. Other baseline characteristics are shown in **Supplementary Table 3**.

**Table 2:** Baseline characteristics of participants with glycemic abnormalities.

Variable	Total (n=10089)	HUA group (n=2735)	non-HUA group (n=7354)	P-value
Age	59.22 ± 13.07	58.22 ± 14.01	59.59 ± 12.68	<0.001
Sex				<0.001
female	5497 (54.5%)	1117 (40.8%)	4380 (59.6%)	
male	4592 (45.5%)	1618 (59.2%)	2974 (40.4%)	
Hypertension				0.600
Yes	5915 (58.6%)	1615 (59.0%)	4300 (58.5%)	
No	4174 (41.4%)	1120 (41.0%)	3054 (41.5%)	
Smoking history				0.030
Yes	3067 (30.4%)	876 (32.0%)	2191 (29.8%)	
No	7022 (69.6%)	1859 (68.0%)	5163 (70.2%)	
Alcohol Drinking				0.452
Yes	4200 (41.6%)	1122 (41.0%)	3078 (41.9%)	
No	5889 (58.4%)	1613 (59.0%)	4276 (58.1%)	
Fatty liver disease				<0.001
Yes	1245 (12.3%)	433 (15.8%)	812 (11.0%)	
No	8844 (87.7%)	2302 (84.2%)	6542 (89.0%)	
BMI (kg/m^2^)	27.28 ± 3.94	27.58 ± 3.73	27.17 ± 4.01	<0.001
Immune-inflammatory indices				
NLR	1.77 (1.39, 2.29)	1.77 (1.41, 2.29)	1.77 (1.39, 2.29)	0.395
MLR	0.20 (0.16, 0.25)	0.21 (0.17, 0.26)	0.20 (0.16, 0.25)	<0.001
PLR	118.95 (95.03, 149.07)	112.15 (91.19, 137.94)	122.00 (96.91, 152.68)	<0.001
SIRI	0.71 (0.51, 1.00)	0.77 (0.55, 1.07)	0.69 (0.49, 0.97)	<0.001
SII	415.15 (309.01, 561.36)	411.70 (311.06, 559.93)	416.46 (307.60, 561.57)	0.693
PIV	165.86 (112.90, 241.95)	174.94 (122.64, 257.13)	162.01 (110.30, 236.46)	<0.001
Immune-lipid indices				
WHR	4.41 (3.30, 5.71)	5.02 (3.91, 6.27)	4.18 (3.15, 5.45)	<0.001
NHR	2.51 (1.82, 3.37)	2.84 (2.14, 3.73)	2.39 (1.74, 3.21)	<0.001
LHR	1.41 (1.04, 1.85)	1.60 (1.21, 2.04)	1.33 (0.99, 1.77)	<0.001
MHR	0.28 (0.21, 0.38)	0.33 (0.25, 0.43)	0.27 (0.20, 0.36)	<0.001
PHR	168.70 (130.60, 213.39)	180.00 (140.88, 225.32)	163.69 (127.25, 208.33)	<0.001
Metabolic composite indices				
RC	0.71 (0.50, 1.03)	0.76 (0.57, 1.09)	0.69 (0.48, 1.00)	<0.001
NHHR	2.78 (2.05, 3.62)	3.15 (2.41, 3.92)	2.63 (1.95, 3.45)	<0.001
AIP	0.09 ± 0.33	0.20 ± 0.30	0.05 ± 0.33	<0.001
TyG	8.82 (8.44, 9.37)	9.04 (8.67, 9.58)	8.74 (8.37, 9.26)	<0.001
METS-IR	41.57 (35.95, 45.97)	43.28 (38.31, 47.39)	40.81 (35.14, 45.30)	<0.001
CHG	5.26 (5.02, 5.51)	5.36 (5.16, 5.58)	5.21 (4.97, 5.47)	<0.001

Significant differences were observed between the HUA and non-HUA groups in several demographic and clinical characteristics. Participants with HUA were slightly younger than those without HUA (58.22 ± 14.01 vs. 59.59 ± 12.68 years, P < 0.001). The proportion of men was higher in the HUA group than in the non-HUA group (59.2% vs. 40.4%, P < 0.001). The HUA group also had a higher BMI (27.58 ± 3.73 vs. 27.17 ± 4.01 kg/m², P < 0.001) and a higher frequency of fatty liver disease (15.8% vs. 11.0%, P < 0.001).

For immune-inflammatory indices, the HUA group had higher levels of MLR [0.21 (0.17, 0.26) vs. 0.20 (0.16, 0.25), P < 0.001], SIRI [0.77 (0.55, 1.07) vs. 0.69 (0.49, 0.97), P < 0.001], and PIV [174.94 (122.64, 257.13) vs. 162.01 (110.30, 236.46), P < 0.001], whereas PLR was lower [112.15 (91.19, 137.94) vs. 122.00 (96.91, 152.68), P < 0.001]. No significant differences were found in NLR or SII.

For immune–lipid indices, all five indicators were significantly higher in the HUA group, including WHR [5.02 (3.91, 6.27) vs. 4.18 (3.15, 5.45), P < 0.001], NHR [2.84 (2.14, 3.73) vs. 2.39 (1.74, 3.21), P < 0.001], LHR [1.60 (1.21, 2.04) vs. 1.33 (0.99, 1.77), P < 0.001], MHR [0.33 (0.25, 0.43) vs. 0.27 (0.20, 0.36), P < 0.001], and PHR [180.00 (140.88, 225.32) vs. 163.69 (127.25, 208.33), P < 0.001].

The metabolic composite indices were significantly higher in the HUA group than in the non-HUA group, including RC [0.76 (0.57, 1.09) vs. 0.69 (0.48, 1.00), P < 0.001], NHHR [3.15 (2.41, 3.92) vs. 2.63 (1.95, 3.45), P < 0.001], AIP (0.20 ± 0.30 vs. 0.05 ± 0.33, P < 0.001), TyG index [9.04 (8.67, 9.58) vs. 8.74 (8.37, 9.26), P < 0.001], METS-IR [43.28 (38.31, 47.39) vs. 40.81 (35.14, 45.30), P < 0.001], and CHG [5.36 (5.16, 5.58) vs. 5.21 (4.97, 5.47), P < 0.001]. Overall, participants with HUA showed more pronounced immune–lipid and metabolic abnormalities than those without HUA.

### 3.3 Associations between composite indices and hyperuricemia

The associations between each composite index and HUA were further evaluated using logistic regression models (**Figure 2**). In the unadjusted model, MLR, SIRI, PIV, WHR, NHR, LHR, MHR, PHR, NHHR, AIP, TyG index, METS-IR, and CHG were positively associated with HUA, whereas PLR was inversely associated with HUA. After adjustment for age and sex, the associations remained significant for PLR, SIRI, PIV, WHR, NHR, LHR, MHR, PHR, AIP, TyG index, METS-IR, and CHG.

**Figure 2:**
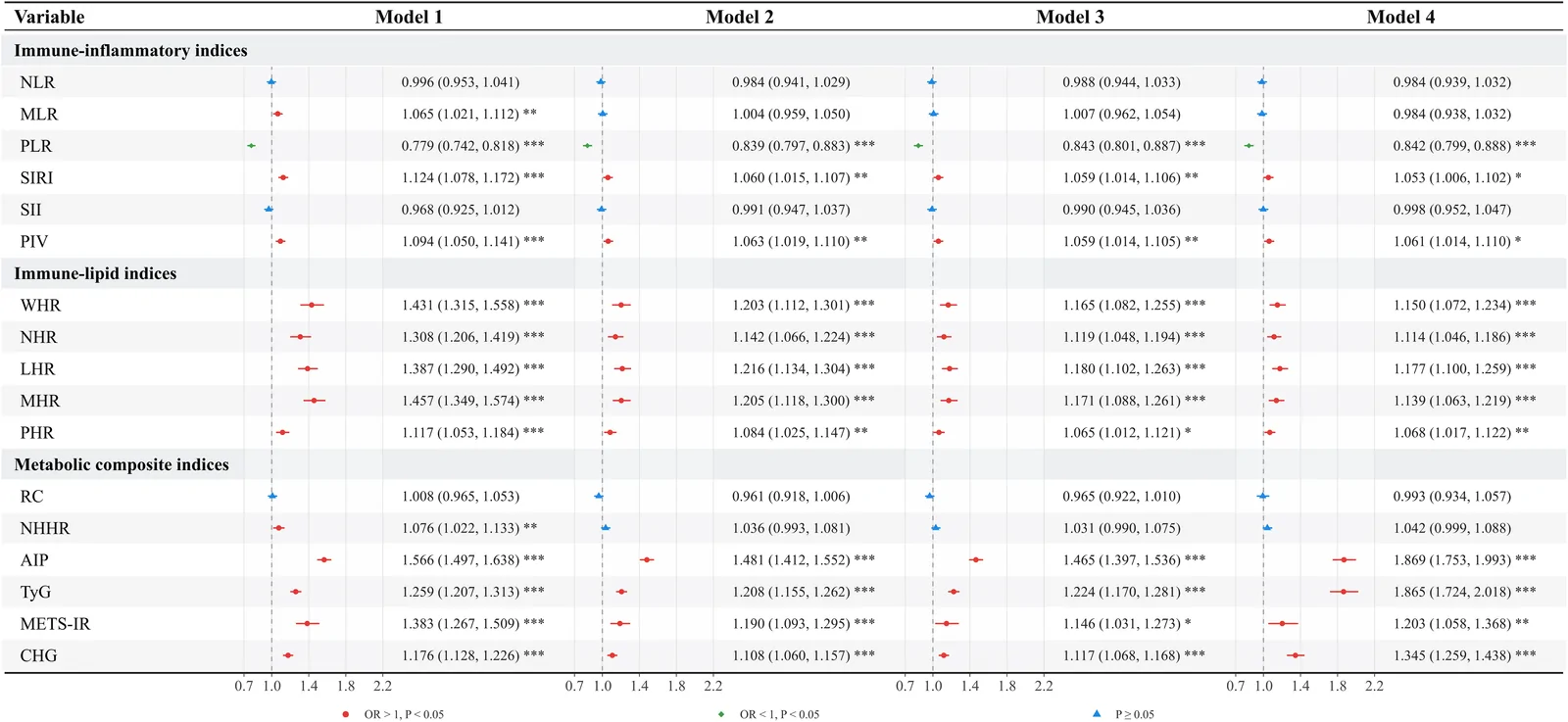
Multivariable logistic regression analysis of the associations between composite indices and hyperuricemia. Model 1, no covariates were adjusted. Model 2, adjusted for age and sex. Model 3, adjusted for age, sex, BMI, hypertension, fatty liver disease, smoking history, and alcohol drinking. Model 4, adjusted for age, sex, BMI, hypertension, fatty liver disease, smoking history, alcohol drinking, glycemic status, and eGFR. OR, odds ratio; CI, confidence interval. *p < 0.05; **p < 0.01; ***p < 0.001. A two-sided p value < 0.05 was considered statistically significant.

In Model 3, after controlling for age, sex, BMI, hypertension, fatty liver disease, smoking history, and alcohol drinking, several composite indices remained significantly associated with HUA. Among immune-inflammatory indices, PLR was inversely associated with HUA (OR = 0.843, 95% CI: 0.801–0.887, P < 0.001), whereas SIRI (OR = 1.059, 95% CI: 1.014–1.106, P < 0.01) and PIV (OR = 1.059, 95% CI: 1.014–1.105, P < 0.01) were positively associated with HUA; in contrast, NLR, MLR, and SII were not significant after adjustment. For immune–lipid composite indices, WHR (OR = 1.165, 95% CI: 1.082–1.255, P < 0.001), NHR (OR = 1.119, 95% CI: 1.048–1.194, P < 0.001), LHR (OR = 1.180, 95% CI: 1.102–1.263, P < 0.001), MHR (OR = 1.171, 95% CI: 1.088–1.261, P < 0.001), and PHR (OR = 1.065, 95% CI: 1.012–1.121, P < 0.05) were all positively associated with HUA. Among metabolic indices, AIP showed the strongest positive association with HUA (OR = 1.465, 95% CI: 1.397–1.536, P < 0.001), followed by TyG index (OR = 1.224, 95% CI: 1.170–1.281, P < 0.001), METS-IR (OR = 1.146, 95% CI: 1.031–1.273, P < 0.05), and CHG (OR = 1.117, 95% CI: 1.068–1.168, P < 0.001), whereas RC and NHHR were not significantly associated with HUA.

After further adjustment for eGFR and glycemic status in Model 4, most of these associations remained significant, indicating that the observed associations were generally robust to additional adjustment for renal function. Overall, immune–lipid and metabolic composite indices appeared to show better associations with HUA than traditional immune-inflammatory indices in individuals with glycemic abnormalities. The full logistic regression results corresponding to **Figure 2** are provided in **Supplementary Table 4**.

### 3.4 Restricted cubic spline analysis

Restricted cubic spline analyses were performed to further explore the potential nonlinear associations between selected composite indices and HUA (**Figure 3**). Several immune-inflammatory, immune–lipid, and metabolic composite indices showed nonlinear associations with the odds of HUA. Among immune-inflammatory indices, PLR, SIRI, and PIV exhibited distinct nonlinear patterns. PLR showed an inverse association with HUA at higher levels, whereas SIRI and PIV were positively associated with HUA below their corresponding inflection points, followed by attenuation or plateauing above these thresholds. For immune–lipid composite indices, WHR, NHR, LHR, MHR, and PHR showed broadly similar nonlinear patterns, with the odds of HUA increasing at lower-to-moderate levels and then reaching a plateau or showing attenuation at higher levels. Nonlinear patterns were also observed for metabolic composite indices, including AIP, TyG index, METS-IR, and CHG, although the curve shapes differed across indices. To further quantify these nonlinear associations, two-piecewise logistic regression models were used to identify inflection points and estimate the associations on both sides of each threshold. The detailed inflection points from the two-piecewise logistic regression analyses are shown in **Supplementary Table 5**. These results suggest nonlinear and threshold-dependent associations between composite indices and HUA among individuals with glycemic abnormalities.

**Figure 3:**
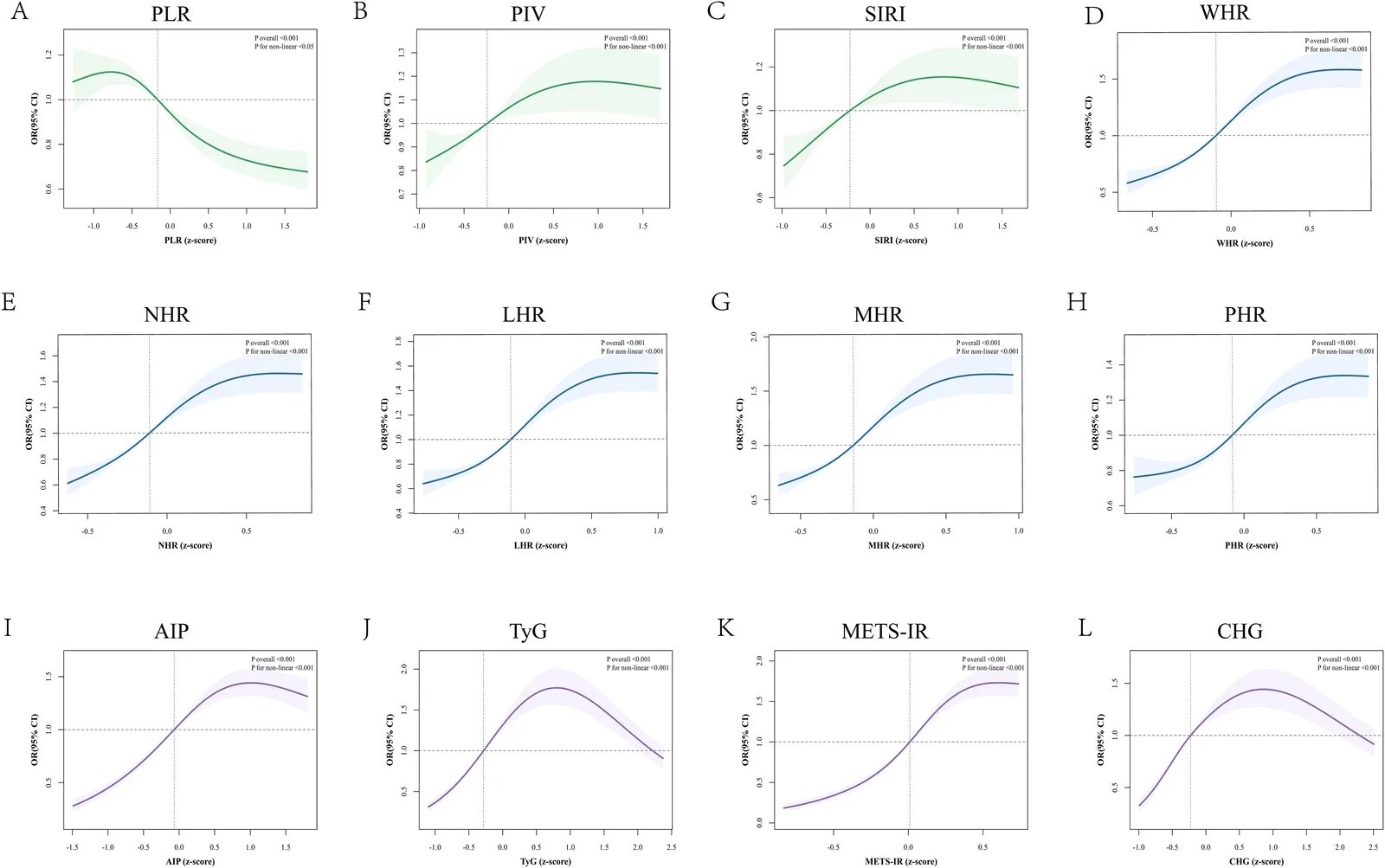
Restricted cubic spline analysis of composite indices **(A)** PLR; **(B)** PIV; **(C)** SIRI; **(D)** WHR; **(E)** NHR; **(F)** LHR; **(G)** MHR; **(H)** PHR; **(I)** AIP; **(J)** TyG; **(K)** METS-IR; **(L)** CHG) in relation to the odds of hyperuricemia among individuals with glycemic abnormalities. The model was adjusted for age, sex, BMI, smoking history, alcohol drinking, hypertension, and fatty liver disease.

### 3.5 Subgroup analysis

Subgroup analyses showed heterogeneous associations between composite indices and HUA across several population subgroups. Sex showed the most consistent modifying effect, with significant interactions observed for all 12 composite indices (**Figure 4**). For SIRI, PIV, WHR, NHR, LHR, MHR, PHR, TyG index, AIP, METS-IR, and CHG, the positive associations with HUA were generally stronger in women than in men. Age and BMI were also important modifiers. Significant interactions with age were observed for many indices, with stronger associations generally found among participants aged <60 years than among those aged ≥60 years, particularly for METS-IR, WHR, NHR, LHR, and MHR. BMI modified the associations for 8 indices, and the associations were consistently stronger among participants with BMI <24 kg/m² than among those with BMI ≥24 kg/m². Significant interactions were also observed for selected indices across hypertension, smoking, alcohol drinking, and fatty liver disease subgroups. Specifically, hypertension modified the associations of LHR, MHR, METS-IR, PLR, PIV, PHR, and SIRI with HUA; smoking modified the associations of WHR, NHR, and LHR; alcohol drinking modified the associations of WHR, NHR, MHR, PHR, and METS-IR; fatty liver disease modified the associations of CHG, NHR, AIP, and WHR; and glycemic status modified the associations of SIRI, PIV, WHR, NHR, LHR, MHR, PHR, AIP, TyG index, METS-IR, and CHG, with these associations generally being more pronounced among participants with prediabetes than among those with diabetes. eGFR modified the associations of WHR, NHR, LHR, PHR, and METS-IR, with stronger associations generally observed at lower eGFR levels. The specific ORs and 95% CIs are presented in the **Supplementary Table 6**.

**Figure 4:**
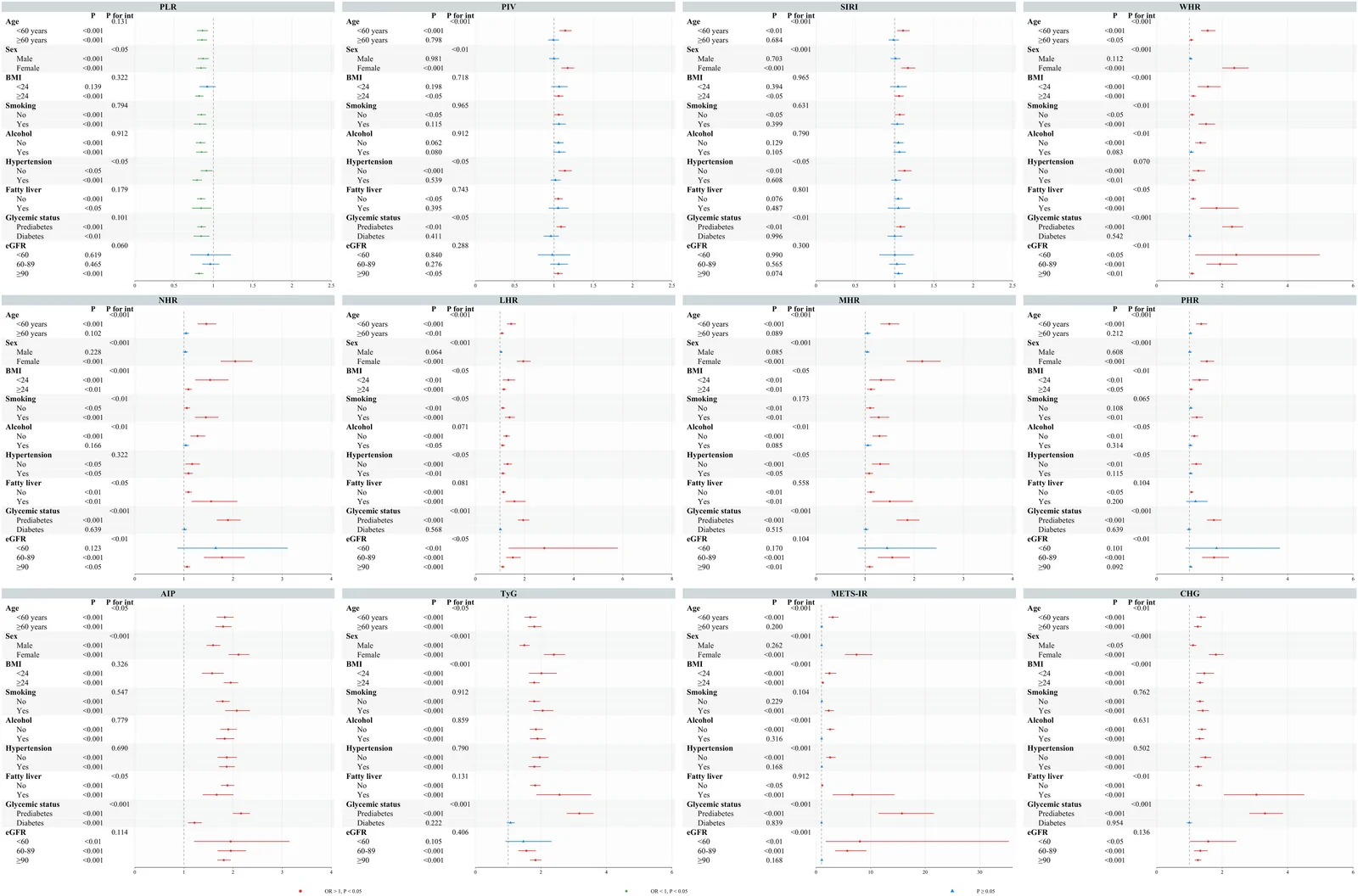
Subgroup analyses of the associations between composite indices and hyperuricemia among individuals with glycemic abnormalities. Odds ratios and 95% confidence intervals were estimated per 1-SD increase in each Z-score–standardized composite index. Subgroup analyses were conducted according to age, sex, BMI, hypertension, fatty liver disease, smoking history, alcohol drinking, glycemic status, and eGFR. Models were adjusted for age, sex, BMI, hypertension, fatty liver disease, smoking history, and alcohol drinking, glycemic status, and eGFR, except for the corresponding stratification variable. Raw P values for interaction were adjusted using the Benjamini–Hochberg false discovery rate method.

## 4 Discussion

This study suggests that among Chinese adults with glycemic abnormalities, hyperuricemia is more strongly associated with combined immune–lipid and glucose–lipid metabolic dysregulation than with isolated inflammatory burden. AIP showed the strongest association with hyperuricemia. Several of these associations were nonlinear and exhibited heterogeneity across sex, age, and BMI subgroups, with additional effect modification observed according to glycemic status and eGFR.

Among glucose–lipid metabolic indices, AIP was one of the strongest indicators associated with HUA after adjustment for confounding factors, which is consistent with previous studies reporting an independent association between AIP and SUA levels as well as HUA risk (30). Moreover, a significant nonlinear association was observed between AIP and HUA, with the OR for HUA increasing at low-to-moderate AIP levels and then reaching a plateau at higher levels. In addition to reflecting atherogenic dyslipidemia, AIP has also been associated with renal dysfunction under conditions of glycemic abnormalities, including proteinuria, elevated uric acid, increased creatinine, and decreased estimated glomerular filtration rate (eGFR) (31–33). Consistent with this, TyG, METS-IR, and CHG, which integrate glucose–lipid metabolic information, were also associated with HUA and showed nonlinear patterns, although their associations were weaker than the association observed for AIP. TyG and CHG exhibited approximately inverted U-shaped associations, which may reflect metabolic heterogeneity among individuals with severe glycemic abnormalities, treatment-related effects, or unstable estimates due to sparse data in the upper range. In contrast, METS-IR showed an approximately “S”-shaped nonlinear association with HUA, with the OR increasing and then plateauing at higher METS-IR levels, suggesting a potential threshold or saturation effect. By comparison, RC and NHHR, which mainly reflect lipid burden, showed no significant association with HUA. This may suggest that lipid indices incorporating broader information related to atherogenic dyslipidemia and insulin resistance capture metabolic features more closely associated with HUA than indices primarily reflecting cholesterol burden. Notably, AIP, composed of TG/HDL-C, may more sensitively reflect lipid abnormalities characterized by high TG and low HDL-C. This atherogenic dyslipidemic phenotype may be closely linked to insulin resistance, oxidative stress, endothelial dysfunction, and lipid-related kidney injury, which collectively may contribute to increased uric acid production and reduced renal urate excretion (13, 34–36). Accordingly, the weaker associations observed for RC and NHHR may reflect their predominant characterization of remnant lipoprotein cholesterol or non-HDL cholesterol burden rather than broader metabolic dysregulation.

In addition to metabolic composite indices, another important finding of this study was that immune–lipid composite indices, including WHR, NHR, LHR, MHR, and PHR, were all positively associated with HUA after adjustment for confounding factors. All showed approximately S-shaped nonlinear associations, with the OR increasing rapidly at low-to-moderate levels and then reaching a plateau at higher levels, suggesting a potential threshold or saturation effect between immune–lipid imbalance and HUA risk. Unlike traditional immune-inflammatory indices, these indices combine peripheral blood cell counts with HDL-C, reflecting not only the burden of circulating inflammatory cells but also the relative imbalance between immune-inflammatory activation and HDL-C-mediated protective capacity. This suggests that HDL-C-related inflammatory–lipid imbalance may play an important role in HUA among individuals with glycemic abnormalities. HDL-C has anti-inflammatory, antioxidant, endothelial-protective, and reverse cholesterol transport functions, whereas both the quantity and function of HDL-C may be impaired under conditions of glycemic abnormalities, insulin resistance, and metabolic syndrome (37, 38). Therefore, higher immune–lipid indices may indicate enhanced inflammatory cell activity, accompanied by weakened HDL-C-mediated anti-inflammatory and vascular protective effects (39, 40). Compared with traditional immune-inflammatory indices, immune–lipid indices showed more consistent associations with HUA. These indices may therefore provide a more integrated assessment of inflammatory burden and HDL-C-related protective capacity than conventional single inflammatory markers. This suggests that HUA in individuals with glycemic abnormalities may not be driven solely by systemic inflammation, but may represent a pathological state involving both increased inflammatory burden and reduced lipid-related protective capacity.

Regarding traditional immune-inflammatory indices, this study found that their associations with HUA were less consistent than those of immune–lipid composite indices and metabolic composite indices. After adjustment for confounding factors, SIRI and PIV were positively associated with HUA, PLR was negatively associated with HUA, whereas NLR, MLR, and SII showed no significant associations. This suggests that composite inflammatory indices involving neutrophils, monocytes, and platelets may reflect certain aspects of the inflammatory background associated with urate metabolic abnormalities, whereas individual blood cell-derived ratios may be insufficient to fully capture the complex metabolic–inflammatory profile of HUA in individuals with glycemic abnormalities. Notably, PLR was negatively associated with HUA. PLR reflects the relative balance between platelet count and lymphocyte count. Although it is commonly regarded as a marker of systemic inflammation, its level may be influenced by multiple factors, including metabolic status, immune cell redistribution, medication use, underlying diseases, and chronic low-grade inflammation (41, 42). Therefore, its biological meaning may vary across different pathological and metabolic contexts (43, 44). Currently, direct evidence regarding the relationship between PLR and HUA remains limited and inconsistent. Previous studies have also suggested that the associations between blood cell-derived inflammatory indices and HUA may differ according to specific biomarkers and clinical contexts (45, 46). Therefore, the negative association between PLR and HUA observed in this study should be interpreted with caution and should not be regarded as evidence that PLR has a protective effect against HUA. Further prospective and mechanistic studies are needed to clarify the biological basis and clinical significance of this finding.

Exploratory subgroup analyses showed that the associations between composite indices and HUA varied across population subgroups, with sex, age, and BMI showing the most prominent heterogeneity among demographic and anthropometric factors. Significant interactions by sex were observed for all 12 indices, and the positive associations of most immune–lipid and metabolic indices with HUA were generally stronger in women than in men. Similar sex-specific patterns have been reported in studies of TyG-related indices, SIRI, and uric acid-related metabolic markers. This sex-specific pattern may be related to differences in estrogen status, postmenopausal changes in fat distribution, reduced uric acid excretion, insulin resistance, and inflammatory activation. Experimental and clinical evidence suggests that estradiol may promote uric acid excretion, whereas menopause is associated with visceral fat accumulation, adipose tissue inflammation, and reduced insulin sensitivity (47–49). Interactions by age were also observed, with stronger associations generally found among participants aged <60 years. Similar age-dependent associations have been reported for CHG- and METS-IR-related outcomes, with stronger associations observed in younger or non-elderly individuals (28, 50). In younger individuals, elevated composite indices may reflect earlier and more active metabolic deterioration, whereas in older adults, multimorbidity, medication use, altered renal function, and age-related physiological heterogeneity may weaken the explanatory value of a single composite biomarker. Similarly, stronger associations were observed among participants with BMI <24 kg/m². This finding is noteworthy because, particularly in Chinese and other Asian populations, normal BMI does not necessarily exclude visceral fat accumulation, fatty liver disease, insulin resistance, or metabolic dysfunction (51, 52). Therefore, elevated composite indices in non-overweight individuals may reveal hidden metabolic risk that is not adequately captured by BMI alone. Beyond these demographic and anthropometric factors, glycemic status modified the associations for most indices, with generally stronger associations observed among participants with prediabetes than among those with diabetes. This attenuation in established diabetes may reflect greater metabolic heterogeneity and the influence of disease duration and glucose-lowering treatment. For glucose-containing indices such as TyG, METS-IR, and CHG, differences in the distribution and clinical relevance of FPG across glycemic strata may also partly contribute to the observed heterogeneity. Regarding eGFR, effect modification was observed for fewer indices, mainly for immune–lipid indices and METS-IR, with generally stronger associations observed at lower eGFR levels. Given the central role of renal urate excretion in HUA, reduced renal function may partly contribute to this pattern.

This study has several notable strengths and innovations. Unlike studies conducted in the general population, it specifically focused on adults with glycemic abnormalities, a clinically relevant group characterized by a high cardiometabolic risk burden. Rather than examining a single indicator, the study simultaneously evaluated multiple categories of composite indices—including immune-inflammatory, immune–lipid, and metabolic indices—within the same population, allowing for a more integrated comparison. In addition, the use of multivariable logistic regression, restricted cubic spline analysis, and subgroup analysis enabled a more comprehensive assessment of not only linear associations but also potential nonlinear relationships and population heterogeneity. Because these indices can be derived from routine health examination data, they may have potential value for assisting metabolic risk assessment in individuals with glycemic abnormalities. However, given the cross-sectional design of this study, they should currently be regarded as adjunctive markers for risk identification or metabolic phenotyping rather than diagnostic or predictive tools for HUA. Several limitations should also be considered. The cross-sectional design precludes any inference of causal relationships between composite indices and HUA. As the study was conducted at a single center using health examination data, selection bias may be present, and the generalizability of the findings to other regions, ethnic groups, and clinical populations remains uncertain. Although multiple covariates were adjusted for, residual confounding cannot be excluded due to the lack of information on dietary purine intake, physical activity, diabetes duration, and glucose-lowering treatment. In addition, the potential influence of urate-lowering drugs, diuretics, Sodium–glucose cotransporter 2 (SGLT2) inhibitors, and lipid-lowering medications was not fully accounted for. It should be noted that several metabolic composite indices share common components, including TG, HDL-C, FPG, and BMI, resulting in inherent correlations and overlapping metabolic information among these indices. As each composite index was entered separately into the regression models rather than simultaneously, the influence of conventional multicollinearity between the indices may have been limited to some extent. Nevertheless, the shared components and resulting overlap in metabolic information should still be considered when interpreting and comparing their associations with HUA. The absence of data on menopausal status and sex hormone levels also restricts a more detailed explanation of the observed sex-specific differences. Furthermore, the findings have not been externally validated. Future prospective, multicenter studies incorporating more detailed information on medication use, renal function, hormonal status, and lifestyle factors are needed to confirm these results and to better elucidate the underlying mechanisms.

## 5 Conclusion

In summary, among Chinese adults with abnormal blood glucose levels, all three categories of indices were associated with HUA. Among the metabolic composite indices, AIP showed the most significant association with HUA; TyG, METS-IR, and CHG were also associated with HUA, whereas the associations of RC and NHHR with HUA were not statistically significant. Among the immune–lipid indices, all indicators, including WHR, NHR, LHR, MHR, and PHR, showed stable and consistent associations with HUA. Among the conventional immune-inflammatory indicators, PLR, PIV, and SIRI were associated with HUA. Further analyses showed that these associations were nonlinear, and subgroup differences indicated that they may be influenced by individual characteristics and metabolic background.

## Data Availability

The original contributions presented in the study are included in the article/**Supplementary Material**. Further inquiries can be directed to the corresponding authors.
