# Supplementary material for: Comparative associations of immune-inflammatory, immune-lipid, and metabolic composite indices with hyperuricemia among Chinese adults with glycemic abnormalities: a health examination-based cross-sectional study
Source: Front Endocrinol (Lausanne). 2026 Sep 14;17:1927643. doi: 10.3389/fendo.2026.1927643 (PMC13616725; doi:10.3389/fendo.2026.1927643)
Supplement: Supplementary file 1 [file SupplementaryFile1.docx]

***Supplementary materials***

[Supplementary Table S1. STROBE checklist for cross-sectional studies	2](#_Toc234006096)

[Supplementary Table S2. Coding scheme for variables included in the logistic regression models	5](#_Toc234006097)

[Supplementary Table S3. Additional baseline characteristics of participants	6](#_Toc234006098)

[Supplementary Table S4. Full logistic regression results for the associations between composite indices and hyperuricemia	7](#_Toc234006099)

[Supplementary Table S5. Results of the two-piecewise logistic regression models	10](#_Toc234006100)

[Supplementary Table S6. Subgroup analyses of the associations between composite indices and hyperuricemia among individuals with glycemic abnormalities	11](#_Toc234006101)

**Supplementary Table S1. STROBE checklist for cross-sectional studies**

STROBE Statement—Checklist of items that should be included in reports of ***cross-sectional studies***

|  | **Item No** | **Recommendation** | Page |
| --- | --- | --- | --- |
| **Title and abstract** | 1 | (*a*) Indicate the study’s design with a commonly used term in the title or the abstract | 2-3 |
|  |  | (*b*) Provide in the abstract an informative and balanced summary of what was done and what was found | 2-3 |
| **Introduction** | | |  |
| Background/rationale | 2 | Explain the scientific background and rationale for the investigation being reported | 4-5 |
| Objectives | 3 | State specific objectives, including any prespecified hypotheses | 4-5 |
| **Methods** | | |  |
| Study design | 4 | Present key elements of study design early in the paper | 5 |
| Setting | 5 | Describe the setting, locations, and relevant dates, including periods of recruitment, exposure, follow-up, and data collection | 5 |
| Participants | 6 | (*a*) Give the eligibility criteria, and the sources and methods of selection of participants | 7 |
| Variables | 7 | Clearly define all outcomes, exposures, predictors, potential confounders, and effect modifiers. Give diagnostic criteria, if applicable | 6-7 |
| Data sources/ measurement | 8* | For each variable of interest, give sources of data and details of methods of assessment (measurement). Describe comparability of assessment methods if there is more than one group | 5-6 |
| Bias | 9 | Describe any efforts to address potential sources of bias | 6-7 |
| Study size | 10 | Explain how the study size was arrived at | 7-8 |
| Quantitative variables | 11 | Explain how quantitative variables were handled in the analyses. If applicable, describe which groupings were chosen and why | 7-9 |
| Statistical methods | 12 | (*a*) Describe all statistical methods, including those used to control for confounding | 7-9 |
|  |  | (*b*) Describe any methods used to examine subgroups and interactions | 7-9 |
|  |  | (*c*) Explain how missing data were addressed | 7-9 |
|  |  | (*d*) If applicable, describe analytical methods taking account of sampling strategy | 7-9 |
|  |  | (*e*) Describe any sensitivity analyses | 7-9 |
| **Results** | | |  |
| Participants | 13* | (a) Report numbers of individuals at each stage of study—eg numbers potentially eligible, examined for eligibility, confirmed eligible, included in the study, completing follow-up, and analysed | 7-8 |
|  |  | (b) Give reasons for non-participation at each stage | 7-8 |
|  |  | (c) Consider use of a flow diagram | 7-8 |
| Descriptive data | 14* | (a) Give characteristics of study participants (eg demographic, clinical, social) and information on exposures and potential confounders | 9-10 |
|  |  | (b) Indicate number of participants with missing data for each variable of interest | 9-10 |
| Outcome data | 15* | Report numbers of outcome events or summary measures | 10-11 |
| Main results | 16 | (*a*) Give unadjusted estimates and, if applicable, confounder-adjusted estimates and their precision (eg, 95% confidence interval). Make clear which confounders were adjusted for and why they were included | 11-18 |
|  |  | (*b*) Report category boundaries when continuous variables were categorized | 11-18 |
|  |  | (*c*) If relevant, consider translating estimates of relative risk into absolute risk for a meaningful time period | 11-18 |
| Other analyses | 17 | Report other analyses done—eg analyses of subgroups and interactions, and sensitivity analyses | 19-20 |
| **Discussion** | | |  |
| Key results | 18 | Summarise key results with reference to study objectives | 21-24 |
| Limitations | 19 | Discuss limitations of the study, taking into account sources of potential bias or imprecision. Discuss both direction and magnitude of any potential bias | 21-24 |
| Interpretation | 20 | Give a cautious overall interpretation of results considering objectives, limitations, multiplicity of analyses, results from similar studies, and other relevant evidence | 21-24 |
| Generalisability | 21 | Discuss the generalisability (external validity) of the study results | 21-24 |
| **Other information** | | |  |
| Funding | 22 | Give the source of funding and the role of the funders for the present study and, if applicable, for the original study on which the present article is based | 27 |

**Supplementary Table S2. Coding scheme for variables included in the logistic regression models**

| **Variable** | **Coding** |
| --- | --- |
| Sex | Male=0，Female=1 |
| Smoking history | No=0，Yes=1 |
| Alcohol drinking | No=0，Yes=1 |
| Hypertension | No=0，Yes=1 |
| Fatty liver | No=0，Yes=1 |
| **Glycemic status** | **Prediabetes=0，Diabetes=1** |

**Supplementary Table S3. Additional baseline characteristics of participants**

| Variable | Total  (n=10089) | HUA group  (n=2735) | non-HUA group  (n=7354) | P-value |
| --- | --- | --- | --- | --- |
| WBC (10^9^/L) | 6.34 ± 1.65 | 6.66 ± 1.64 | 6.22 ± 1.64 | <0.001 |
| Neut (10^9^/L) | 3.71 ± 1.27 | 3.88 ± 1.24 | 3.64 ± 1.27 | <0.001 |
| Lymph (10^9^/L) | 2.04 ± 0.60 | 2.13 ± 0.61 | 2.00 ± 0.60 | <0.001 |
| Mono (10^9^/L) | 0.40 (0.33, 0.49) | 0.43 (0.35, 0.52) | 0.39 (0.32, 0.48) | <0.001 |
| Eos (10^9^/L) | 0.11 (0.07, 0.18) | 0.12 (0.08, 0.20) | 0.10 (0.06, 0.17) | <0.001 |
| Baso (10^9^/L) | 0.03 (0.02, 0.04) | 0.03 (0.03, 0.05) | 0.03 (0.02, 0.04) | <0.001 |
| RBC (10^12^/L) | 4.70 ± 0.47 | 4.82 ± 0.47 | 4.65 ± 0.46 | <0.001 |
| Hgb (g/L) | 141.67 ± 14.71 | 145.55 ± 13.86 | 140.23 ± 14.76 | <0.001 |
| PLT (10^9^/L) | 239.78 ± 58.71 | 238.09 ± 58.67 | 240.41 ± 58.71 | 0.077 |
| TBIL (μmol/L) | 15.78 ± 5.67 | 15.82 ± 5.96 | 15.76 ± 5.56 | 0.617 |
| ALT (U/L) | 20.30 (14.90, 29.70) | 24.70 (17.30, 37.70) | 19.10 (14.20, 27.00) | <0.001 |
| AST (U/L) | 21.60 (18.20, 26.38) | 23.30 (19.40, 29.50) | 21.00 (17.90, 25.30) | <0.001 |
| BUN (mmol/L) | 4.98 (4.22, 5.85) | 5.14 (4.36, 6.00) | 4.92 (4.19, 5.80) | <0.001 |
| Scr (μmol/L) | 64.00 (55.00, 74.10) | 71.10 (61.00, 81.00) | 61.30 (53.50, 71.00) | <0.001 |
| TC (mmol/L) | 5.36 (4.56, 6.28) | 5.44 (4.63, 6.35) | 5.33 (4.53, 6.24) | <0.001 |
| TG (mmol/L) | 1.58 (1.09, 2.67) | 1.94 (1.36, 3.23) | 1.46 (1.03, 2.41) | <0.001 |
| HDL-C (mmol/L) | 1.38 (1.17, 1.67) | 1.27 (1.10, 1.52) | 1.42 (1.20, 1.71) | <0.001 |
| LDL-C (mmol/L) | 2.80 ± 0.99 | 2.91 ± 1.03 | 2.76 ± 0.97 | <0.001 |

**Supplementary Table S4. Full logistic regression results for the associations between composite indices and hyperuricemia**

**Model 1**

| **Variable** | **β** | **S.E** | **Z** | **P** | **OR (95%CI)** |
| --- | --- | --- | --- | --- | --- |
| **Immune-inflammatory indices** | | |  |  |  |
| **NLR** | -0.0042 | 0.0225 | -0.185 | 0.853227 | 0.996 (0.953, 1.041) |
| **MLR** | 0.0633 | 0.0219 | 2.8972 | <0.01 | 1.065 (1.021, 1.112) |
| **PLR** | -0.2497 | 0.0251 | -9.968 | <0.001 | 0.779 (0.742, 0.818) |
| **SIRI** | 0.1166 | 0.0213 | 5.4793 | <0.001 | 1.124 (1.078, 1.172) |
| **SII** | -0.0327 | 0.0229 | -1.425 | 1.54E-01 | 0.968 (0.925, 1.012) |
| **PIV** | 0.0903 | 0.0213 | 4.2439 | <0.001 | 1.094 (1.050, 1.141) |
| **Immune-lipid indices** | |  |  |  |  |
| **WHR** | 0.3585 | 0.0432 | 8.2928 | <0.001 | 1.431 (1.315, 1.558) |
| **NHR** | 0.2686 | 0.0417 | 6.4474 | <0.001 | 1.308 (1.206, 1.419) |
| **LHR** | 0.3272 | 0.0371 | 8.8212 | <0.001 | 1.387 (1.290, 1.492) |
| **MHR** | 0.3764 | 0.0395 | 9.5342 | <0.001 | 1.457 (1.349, 1.574) |
| **PHR** | 0.1103 | 0.0301 | 3.6655 | <0.001 | 1.117 (1.053, 1.184) |
| **Metabolic composite indices** | | |  |  |  |
| **RC** | 0.0084 | 0.0223 | 0.3758 | 0.707069 | 1.008 (0.965, 1.053) |
| **NHHR** | 0.0735 | 0.0263 | 2.7945 | <0.01 | 1.076 (1.022, 1.133) |
| **AIP** | 0.4483 | 0.023 | 19.4644 | <0.001 | 1.566 (1.497, 1.638) |
| **TyG** | 0.2303 | 0.0214 | 10.7742 | <0.001 | 1.259 (1.207, 1.313) |
| **METS-IR** | 0.3239 | 0.0446 | 7.2627 | <0.001 | 1.383 (1.267, 1.509) |
| **CHG** | 0.1624 | 0.0212 | 7.6633 | <0.001 | 1.176 (1.128, 1.226) |

**Model 2**

| **Variable** | **β** | **S.E** | **Z** | **P** | **OR (95%CI)** |
| --- | --- | --- | --- | --- | --- |
| **Immune-inflammatory indices** | | |  |  |  |
| **NLR** | -0.0161 | 0.023 | -0.6993 | 0.484386 | 0.984 (0.941, 1.029) |
| **MLR** | 0.0036 | 0.023 | 0.1549 | 0.876885 | 1.004 (0.959, 1.050) |
| **PLR** | -0.1757 | 0.0259 | -6.7786 | <0.001 | 0.839 (0.797, 0.883) |
| **SIRI** | 0.0586 | 0.0221 | 2.656 | <0.01 | 1.060 (1.015, 1.107) |
| **SII** | -0.0091 | 0.0233 | -0.3924 | 0.694744 | 0.991 (0.947, 1.037) |
| **PIV** | 0.0614 | 0.0218 | 2.8161 | <0.01 | 1.063 (1.019, 1.110) |
| **Immune-lipid indices** | |  |  |  |  |
| **WHR** | 0.1847 | 0.0402 | 4.5982 | <0.001 | 1.203 (1.112, 1.301) |
| **NHR** | 0.1328 | 0.0354 | 3.7544 | <0.001 | 1.142 (1.066, 1.224) |
| **LHR** | 0.1958 | 0.0357 | 5.4823 | <0.001 | 1.216 (1.134, 1.304) |
| **MHR** | 0.1869 | 0.0386 | 4.8365 | <0.001 | 1.205 (1.118, 1.300) |
| **PHR** | 0.0806 | 0.0287 | 2.8136 | <0.01 | 1.084 (1.025, 1.147) |
| **Metabolic composite indices** | | |  |  |  |
| **RC** | -0.0396 | 0.0232 | -1.7084 | 0.087555 | 0.961 (0.918, 1.006) |
| **NHHR** | 0.0356 | 0.0216 | 1.6458 | 0.099797 | 1.036 (0.993, 1.081) |
| **AIP** | 0.3925 | 0.0241 | 16.2993 | <0.001 | 1.481 (1.412, 1.552) |
| **TyG** | 0.1886 | 0.0227 | 8.3245 | <0.001 | 1.208 (1.155, 1.262) |
| **METS-IR** | 0.1741 | 0.0433 | 4.0224 | <0.001 | 1.190 (1.093, 1.295) |
| **CHG** | 0.1022 | 0.0224 | 4.5542 | <0.001 | 1.108 (1.060, 1.157) |

**Model 3**

| **Variable** | **β** | **S.E** | **Z** | **P** | **OR (95%CI)** |
| --- | --- | --- | --- | --- | --- |
| **Immune-inflammatory indices** | | |  |  |  |
| **NLR** | -0.0125 | 0.0232 | -0.5394 | 0.589589 | 0.988 (0.944, 1.033) |
| **MLR** | 0.0068 | 0.0232 | 0.2921 | 0.770194 | 1.007 (0.962, 1.054) |
| **PLR** | -0.1708 | 0.0261 | -6.5417 | <0.001 | 0.843 (0.801, 0.887) |
| **SIRI** | 0.0575 | 0.0222 | 2.5878 | <0.01 | 1.059 (1.014, 1.106) |
| **SII** | -0.0102 | 0.0234 | -0.4361 | 0.662769 | 0.990 (0.945, 1.036) |
| **PIV** | 0.0571 | 0.022 | 2.5994 | <0.01 | 1.059 (1.014, 1.105) |
| **Immune-lipid indices** | |  |  |  |  |
| **WHR** | 0.1528 | 0.0379 | 4.0311 | <0.001 | 1.165 (1.082, 1.255) |
| **NHR** | 0.112 | 0.0331 | 3.3812 | <0.001 | 1.119 (1.048, 1.194) |
| **LHR** | 0.1655 | 0.0348 | 4.7633 | <0.001 | 1.180 (1.102, 1.263) |
| **MHR** | 0.1582 | 0.0375 | 4.2207 | <0.001 | 1.171 (1.088, 1.261) |
| **PHR** | 0.0632 | 0.026 | 2.4319 | <0.05 | 1.065 (1.012, 1.121) |
| **Metabolic composite indices** | | |  |  |  |
| **RC** | -0.036 | 0.0233 | -1.5461 | 0.122074 | 0.965 (0.922, 1.010) |
| **NHHR** | 0.0309 | 0.021 | 1.47 | 0.141553 | 1.031 (0.990, 1.075) |
| **AIP** | 0.3818 | 0.0243 | 15.7038 | <0.001 | 1.465 (1.397, 1.536) |
| **TyG** | 0.2025 | 0.023 | 8.8078 | <0.001 | 1.224 (1.170, 1.281) |
| **METS-IR** | 0.136 | 0.0538 | 2.53 | <0.05 | 1.146 (1.031, 1.273) |
| **CHG** | 0.1106 | 0.0227 | 4.8692 | <0.001 | 1.117 (1.068, 1.168) |

Model 4

| **Variable** | **beta** | **S.E** | **Z** | **P** | **OR (95%CI)** |
| --- | --- | --- | --- | --- | --- |
| **Immune-inflammatory indices** |  |  |  |  |  |
| **NLR** | -0.0156 | 0.024 | -0.6507 | 0.515 | 0.984 (0.939, 1.032) |
| **MLR** | -0.0159 | 0.0243 | -0.6519 | 0.514 | 0.984 (0.938, 1.032) |
| **PLR** | -0.1719 | 0.027 | -6.3722 | <0.001 | 0.842 (0.799, 0.888) |
| **SIRI** | 0.0516 | 0.0233 | 2.2177 | 0.027 | 1.053 (1.006, 1.102) |
| **SII** | -0.0018 | 0.0243 | -0.0754 | 0.94 | 0.998 (0.952, 1.047) |
| **PIV** | 0.0592 | 0.0231 | 2.5645 | 0.01 | 1.061 (1.014, 1.110) |
| **Immune-lipid indices** |  |  |  |  |  |
| **WHR** | 0.1396 | 0.0359 | 3.8829 | <0.001 | 1.150 (1.072, 1.234) |
| **NHR** | 0.1081 | 0.032 | 3.382 | <0.001 | 1.114 (1.046, 1.186) |
| **LHR** | 0.1626 | 0.0345 | 4.7156 | <0.001 | 1.177 (1.100, 1.259) |
| **MHR** | 0.1298 | 0.035 | 3.7092 | <0.001 | 1.139 (1.063, 1.219) |
| **PHR** | 0.0657 | 0.0251 | 2.6185 | 0.009 | 1.068 (1.017, 1.122) |
| **Metabolic composite indices** |  |  |  |  |  |
| **RC** | -0.0067 | 0.0316 | -0.2135 | 0.831 | 0.993 (0.934, 1.057) |
| **NHHR** | 0.0415 | 0.0218 | 1.9038 | 0.057 | 1.042 (0.999, 1.088) |
| **AIP** | 0.6253 | 0.0327 | 19.0956 | <0.001 | 1.869 (1.753, 1.993) |
| **TyG** | 0.6233 | 0.0401 | 15.5415 | <0.001 | 1.865 (1.724, 2.018) |
| **METS-IR** | 0.1848 | 0.0654 | 2.8252 | 0.005 | 1.203 (1.058, 1.368) |
| **CHG** | 0.2966 | 0.0339 | 8.7499 | <0.001 | 1.345 (1.259, 1.438) |

**Supplementary Table S5. Results of the two-piecewise logistic regression models**

| Index | Mean | SD | Inflection point  (Z-score) | Inflection point  (original) | OR below  (95% CI) | P | OR above  (95% CI) | P |
| --- | --- | --- | --- | --- | --- | --- | --- | --- |
| PLR | 126.534 | 46.295 | -1.27 | 67.757 | 1.97 (0.96–4.04) | 0.066 | 0.83 (0.78–0.87) | <0.001 |
| SIRI | 0.832 | 0.534 | 0.883 | 1.303 | 1.28 (1.17–1.39) | <0.001 | 0.90 (0.83–0.98) | 0.012 |
| PIV | 201.505 | 147.918 | 1.696 | 452.442 | 1.18 (1.11–1.27) | <0.001 | 0.88 (0.79–0.98) | 0.016 |
| WHR | 4.8 | 4.185 | 0.248 | 5.838 | 3.06 (2.60–3.61) | <0.001 | 0.98 (0.93–1.03) | 0.433 |
| NHR | 2.81 | 2.772 | 0.225 | 3.433 | 2.76 (2.32–3.27) | <0.001 | 0.98 (0.93–1.03) | 0.394 |
| LHR | 1.535 | 1.198 | 0.355 | 1.961 | 2.25 (1.97–2.56) | <0.001 | 0.98 (0.93–1.03) | 0.45 |
| MHR | 0.32 | 0.292 | 0.31 | 0.41 | 2.73 (2.34–3.18) | <0.001 | 0.98 (0.93–1.03) | 0.387 |
| PHR | 179.103 | 133.712 | 0.632 | 263.649 | 1.60 (1.43–1.79) | <0.001 | 0.96 (0.90–1.03) | 0.241 |
| AIP | 0.093 | 0.331 | 0.417 | 0.231 | 2.28 (2.08–2.51) | <0.001 | 0.92 (0.84–1.01) | 0.088 |
| TyG | 9.129 | 1.093 | 0.121 | 9.261 | 3.93 (3.40–4.54) | <0.001 | 0.77 (0.72–0.83) | <0.001 |
| METS-IR | 41.401 | 16.429 | 0.317 | 46.608 | 7.26 (5.71–9.24) | <0.001 | 0.91 (0.81–1.03) | 0.155 |
| CHG | 5.426 | 0.732 | 0.096 | 5.496 | 3.77 (3.22–4.40) | <0.001 | 0.80 (0.75–0.85) | <0.001 |

**Supplementary Table S6. Subgroup analyses of the associations between composite indices and hyperuricemia among individuals with glycemic abnormalities**

| **Variable** | **Subgroup** | **OR (95% CI)** | **P** | **FDR-adjusted**  **P for interaction** |
| --- | --- | --- | --- | --- |
| **PLR** | **Age** |  |  | 0.13149291 |
|  | <60 years | 0.86 (0.80-0.93) | 0.00010879 |  |
|  | ≥60 years | 0.85 (0.79-0.92) | 1.75e-05 |  |
|  | **Sex** |  |  | 0.02785096 |
|  | Male | 0.87 (0.80-0.94) | 0.00029715 |  |
|  | Female | 0.84 (0.78-0.91) | 5.61e-06 |  |
|  | **BMI** |  |  | 0.32202531 |
|  | BMI <24 kg/m2 | 0.92 (0.83-1.03) | 0.13909999 |  |
|  | BMI ≥24 kg/m2 | 0.82 (0.77-0.87) | 7.63e-11 |  |
|  | **Smoking** |  |  | 0.79435095 |
|  | No | 0.85 (0.80-0.90) | 3.89e-07 |  |
|  | Yes | 0.83 (0.75-0.91) | 9.62e-05 |  |
|  | **Alcohol** |  |  | 0.91165712 |
|  | No | 0.84 (0.78-0.90) | 4.58e-07 |  |
|  | Yes | 0.85 (0.78-0.92) | 9.56e-05 |  |
|  | **Hypertension** |  |  | 0.03737179 |
|  | No | 0.91 (0.84-0.98) | 0.01867090 |  |
|  | Yes | 0.79 (0.74-0.85) | 1.67e-10 |  |
|  | **Fatty liver** |  |  | 0.17875799 |
|  | No | 0.84 (0.80-0.89) | 4.87e-09 |  |
|  | Yes | 0.84 (0.73-0.97) | 0.01936590 |  |
|  | **Glycemic status** |  |  | 0.10099610 |
|  | Prediabetes | 0.85 (0.80-0.90) | 7.31e-08 |  |
|  | Diabetes | 0.84 (0.75-0.95) | 0.00421246 |  |
|  | **eGFR** |  |  | 0.05984278 |
|  | eGFR <60 | 0.93 (0.71-1.22) | 0.61934384 |  |
|  | eGFR 60-89 | 0.96 (0.86-1.07) | 0.46500604 |  |
|  | eGFR ≥90 | 0.82 (0.77-0.87) | 1.5e-10 |  |
| **PIV** | **Age** |  |  | 0.00077502 |
|  | <60 years | 1.14 (1.07-1.22) | 6.42e-05 |  |
|  | ≥60 years | 0.99 (0.93-1.06) | 0.79768071 |  |
|  | **Sex** |  |  | 0.00147527 |
|  | Male | 1.00 (0.94-1.06) | 0.98138678 |  |
|  | Female | 1.17 (1.10-1.25) | 3.71e-06 |  |
|  | **BMI** |  |  | 0.71775909 |
|  | BMI <24 kg/m2 | 1.06 (0.97-1.17) | 0.19773992 |  |
|  | BMI ≥24 kg/m2 | 1.06 (1.01-1.11) | 0.02929678 |  |
|  | **Smoking** |  |  | 0.96454320 |
|  | No | 1.06 (1.00-1.12) | 0.04499816 |  |
|  | Yes | 1.06 (0.99-1.15) | 0.11475264 |  |
|  | **Alcohol** |  |  | 0.91165712 |
|  | No | 1.06 (1.00-1.12) | 0.06153405 |  |
|  | Yes | 1.07 (0.99-1.14) | 0.08025462 |  |
|  | **Hypertension** |  |  | 0.02958996 |
|  | No | 1.14 (1.06-1.22) | 0.00042981 |  |
|  | Yes | 1.02 (0.96-1.08) | 0.53906591 |  |
|  | **Fatty liver** |  |  | 0.74279785 |
|  | No | 1.05 (1.00-1.11) | 0.03637436 |  |
|  | Yes | 1.05 (0.94-1.18) | 0.39516992 |  |
|  | **Glycemic status** |  |  | 0.02099304 |
|  | Prediabetes | 1.09 (1.03-1.15) | 0.00131247 |  |
|  | Diabetes | 0.96 (0.87-1.06) | 0.41135518 |  |
|  | **eGFR** |  |  | 0.28780549 |
|  | eGFR <60 | 0.98 (0.80-1.20) | 0.84032925 |  |
|  | eGFR 60-89 | 1.06 (0.96-1.17) | 0.27552668 |  |
|  | eGFR ≥90 | 1.05 (1.00-1.11) | 0.04874198 |  |
| **SIRI** | **Age** |  |  | 0.00082059 |
|  | <60 years | 1.11 (1.03-1.19) | 0.00367738 |  |
|  | ≥60 years | 0.99 (0.93-1.05) | 0.68389607 |  |
|  | **Sex** |  |  | 0.00034587 |
|  | Male | 1.01 (0.95-1.07) | 0.70300681 |  |
|  | Female | 1.17 (1.09-1.26) | 3.37e-05 |  |
|  | **BMI** |  |  | 0.96454320 |
|  | BMI <24 kg/m2 | 1.04 (0.95-1.15) | 0.39387503 |  |
|  | BMI ≥24 kg/m2 | 1.06 (1.00-1.11) | 0.03820036 |  |
|  | **Smoking** |  |  | 0.63085470 |
|  | No | 1.06 (1.01-1.13) | 0.03246946 |  |
|  | Yes | 1.03 (0.96-1.12) | 0.39930670 |  |
|  | **Alcohol** |  |  | 0.79043736 |
|  | No | 1.05 (0.99-1.11) | 0.12947831 |  |
|  | Yes | 1.06 (0.99-1.14) | 0.10547666 |  |
|  | **Hypertension** |  |  | 0.04483818 |
|  | No | 1.13 (1.05-1.21) | 0.00174718 |  |
|  | Yes | 1.02 (0.96-1.08) | 0.60785353 |  |
|  | **Fatty liver** |  |  | 0.80075436 |
|  | No | 1.05 (1.00-1.10) | 0.07573552 |  |
|  | Yes | 1.05 (0.92-1.20) | 0.48738174 |  |
|  | **Glycemic status** |  |  | 0.00875710 |
|  | Prediabetes | 1.07 (1.02-1.13) | 0.00807945 |  |
|  | Diabetes | 1.00 (0.91-1.10) | 0.99626439 |  |
|  | **eGFR** |  |  | 0.29963777 |
|  | eGFR <60 | 1.00 (0.81-1.24) | 0.99012812 |  |
|  | eGFR 60-89 | 1.03 (0.93-1.14) | 0.56509465 |  |
|  | eGFR ≥90 | 1.05 (1.00-1.10) | 0.07447767 |  |
| **WHR** | **Age** |  |  | 1.62e-12 |
|  | <60 years | 1.56 (1.36-1.78) | 8.9e-11 |  |
|  | ≥60 years | 1.06 (1.00-1.12) | 0.03767060 |  |
|  | **Sex** |  |  | 1.09e-24 |
|  | Male | 1.04 (0.99-1.08) | 0.11188538 |  |
|  | Female | 2.37 (2.01-2.80) | 2.77e-24 |  |
|  | **BMI** |  |  | 0.00036732 |
|  | BMI <24 kg/m2 | 1.56 (1.26-1.95) | 5.92e-05 |  |
|  | BMI ≥24 kg/m2 | 1.12 (1.05-1.20) | 0.00080864 |  |
|  | **Smoking** |  |  | 0.00216650 |
|  | No | 1.08 (1.02-1.15) | 0.01281759 |  |
|  | Yes | 1.51 (1.28-1.78) | 1.29e-06 |  |
|  | **Alcohol** |  |  | 0.00221842 |
|  | No | 1.34 (1.19-1.50) | 1.64e-06 |  |
|  | Yes | 1.06 (0.99-1.12) | 0.08292807 |  |
|  | **Hypertension** |  |  | 0.07039882 |
|  | No | 1.27 (1.11-1.47) | 0.00085115 |  |
|  | Yes | 1.11 (1.03-1.19) | 0.00767730 |  |
|  | **Fatty liver** |  |  | 0.01792325 |
|  | No | 1.12 (1.05-1.19) | 0.00080586 |  |
|  | Yes | 1.83 (1.34-2.50) | 0.00014790 |  |
|  | **Glycemic status** |  |  | 4.04e-35 |
|  | Prediabetes | 2.30 (2.01-2.63) | 4.31e-34 |  |
|  | Diabetes | 1.01 (0.97-1.06) | 0.54224685 |  |
|  | **eGFR** |  |  | 0.00188537 |
|  | eGFR <60 | 2.43 (1.19-4.97) | 0.01522995 |  |
|  | eGFR 60-89 | 1.93 (1.53-2.45) | 5.34e-08 |  |
|  | eGFR ≥90 | 1.08 (1.02-1.15) | 0.00559546 |  |
| **NHR** | **Age** |  |  | 5.18e-10 |
|  | <60 years | 1.46 (1.28-1.65) | 6.06e-09 |  |
|  | ≥60 years | 1.04 (0.99-1.10) | 0.10228105 |  |
|  | **Sex** |  |  | 7.3e-19 |
|  | Male | 1.03 (0.98-1.07) | 0.22804022 |  |
|  | Female | 2.04 (1.75-2.39) | 1.91e-19 |  |
|  | **BMI** |  |  | 0.00041981 |
|  | BMI <24 kg/m2 | 1.53 (1.24-1.90) | 0.00010378 |  |
|  | BMI ≥24 kg/m2 | 1.09 (1.03-1.16) | 0.00407383 |  |
|  | **Smoking** |  |  | 0.00197683 |
|  | No | 1.06 (1.00-1.12) | 0.04344100 |  |
|  | Yes | 1.45 (1.23-1.70) | 8.51e-06 |  |
|  | **Alcohol** |  |  | 0.00267507 |
|  | No | 1.28 (1.14-1.43) | 2.45e-05 |  |
|  | Yes | 1.04 (0.98-1.10) | 0.16586359 |  |
|  | **Hypertension** |  |  | 0.32202531 |
|  | No | 1.17 (1.03-1.32) | 0.01323059 |  |
|  | Yes | 1.09 (1.02-1.18) | 0.01573930 |  |
|  | **Fatty liver** |  |  | 0.03737179 |
|  | No | 1.09 (1.03-1.16) | 0.00370362 |  |
|  | Yes | 1.55 (1.16-2.08) | 0.00310562 |  |
|  | **Glycemic status** |  |  | 6.21e-24 |
|  | Prediabetes | 1.90 (1.67-2.15) | 1.89e-23 |  |
|  | Diabetes | 1.01 (0.97-1.06) | 0.63881512 |  |
|  | **eGFR** |  |  | 0.00482775 |
|  | eGFR <60 | 1.65 (0.87-3.11) | 0.12311540 |  |
|  | eGFR 60-89 | 1.78 (1.41-2.23) | 8.78e-07 |  |
|  | eGFR ≥90 | 1.06 (1.01-1.12) | 0.01972501 |  |
| **LHR** | **Age** |  |  | 4.79e-10 |
|  | <60 years | 1.46 (1.30-1.64) | 2.82e-10 |  |
|  | ≥60 years | 1.08 (1.02-1.15) | 0.00997624 |  |
|  | **Sex** |  |  | 1.93e-20 |
|  | Male | 1.04 (1.00-1.09) | 0.06381996 |  |
|  | Female | 1.95 (1.69-2.24) | 1.16e-20 |  |
|  | **BMI** |  |  | 0.01792325 |
|  | BMI <24 kg/m2 | 1.35 (1.12-1.61) | 0.00118380 |  |
|  | BMI ≥24 kg/m2 | 1.16 (1.08-1.24) | 3.66e-05 |  |
|  | **Smoking** |  |  | 0.02958996 |
|  | No | 1.12 (1.04-1.20) | 0.00189589 |  |
|  | Yes | 1.39 (1.21-1.60) | 3.42e-06 |  |
|  | **Alcohol** |  |  | 0.07078321 |
|  | No | 1.26 (1.15-1.39) | 2.12e-06 |  |
|  | Yes | 1.11 (1.02-1.20) | 0.01167816 |  |
|  | **Hypertension** |  |  | 0.02958996 |
|  | No | 1.31 (1.16-1.47) | 1.25e-05 |  |
|  | Yes | 1.12 (1.04-1.20) | 0.00233663 |  |
|  | **Fatty liver** |  |  | 0.08141860 |
|  | No | 1.15 (1.07-1.23) | 4.84e-05 |  |
|  | Yes | 1.59 (1.24-2.04) | 0.00026077 |  |
|  | **Glycemic status** |  |  | 1.84e-29 |
|  | Prediabetes | 1.95 (1.74-2.18) | 3.07e-31 |  |
|  | Diabetes | 1.01 (0.97-1.06) | 0.56780744 |  |
|  | **eGFR** |  |  | 0.03170470 |
|  | eGFR <60 | 2.81 (1.36-5.78) | 0.00512701 |  |
|  | eGFR 60-89 | 1.52 (1.26-1.84) | 1.26e-05 |  |
|  | eGFR ≥90 | 1.11 (1.04-1.18) | 0.00089188 |  |
| **MHR** | **Age** |  |  | 3.44e-13 |
|  | <60 years | 1.49 (1.32-1.69) | 3.38e-10 |  |
|  | ≥60 years | 1.05 (0.99-1.10) | 0.08925127 |  |
|  | **Sex** |  |  | 1.03e-22 |
|  | Male | 1.04 (0.99-1.09) | 0.08519727 |  |
|  | Female | 2.16 (1.84-2.54) | 3.55e-21 |  |
|  | **BMI** |  |  | 0.03561244 |
|  | BMI <24 kg/m2 | 1.32 (1.09-1.60) | 0.00438623 |  |
|  | BMI ≥24 kg/m2 | 1.12 (1.04-1.20) | 0.00133226 |  |
|  | **Smoking** |  |  | 0.17260897 |
|  | No | 1.10 (1.03-1.18) | 0.00804662 |  |
|  | Yes | 1.28 (1.10-1.48) | 0.00148465 |  |
|  | **Alcohol** |  |  | 0.00868004 |
|  | No | 1.29 (1.16-1.44) | 5.16e-06 |  |
|  | Yes | 1.06 (0.99-1.12) | 0.08515368 |  |
|  | **Hypertension** |  |  | 0.01334801 |
|  | No | 1.30 (1.14-1.49) | 9.54e-05 |  |
|  | Yes | 1.08 (1.01-1.16) | 0.02036716 |  |
|  | **Fatty liver** |  |  | 0.55798848 |
|  | No | 1.12 (1.04-1.19) | 0.00118417 |  |
|  | Yes | 1.50 (1.15-1.97) | 0.00319196 |  |
|  | **Glycemic status** |  |  | 2.26e-26 |
|  | Prediabetes | 1.86 (1.65-2.10) | 7.84e-24 |  |
|  | Diabetes | 1.02 (0.97-1.06) | 0.51491028 |  |
|  | **eGFR** |  |  | 0.10364205 |
|  | eGFR <60 | 1.45 (0.85-2.45) | 0.16973554 |  |
|  | eGFR 60-89 | 1.55 (1.26-1.90) | 2.72e-05 |  |
|  | eGFR ≥90 | 1.09 (1.03-1.16) | 0.00502903 |  |
| **PHR** | **Age** |  |  | 4.12e-07 |
|  | <60 years | 1.36 (1.21-1.53) | 3.65e-07 |  |
|  | ≥60 years | 1.03 (0.98-1.08) | 0.21163718 |  |
|  | **Sex** |  |  | 1.67e-07 |
|  | Male | 1.01 (0.96-1.06) | 0.60755009 |  |
|  | Female | 1.53 (1.34-1.75) | 3.89e-10 |  |
|  | **BMI** |  |  | 0.00683689 |
|  | BMI <24 kg/m2 | 1.31 (1.09-1.57) | 0.00356003 |  |
|  | BMI ≥24 kg/m2 | 1.05 (1.00-1.10) | 0.03083082 |  |
|  | **Smoking** |  |  | 0.06458033 |
|  | No | 1.04 (0.99-1.09) | 0.10801866 |  |
|  | Yes | 1.22 (1.06-1.41) | 0.00501747 |  |
|  | **Alcohol** |  |  | 0.04070696 |
|  | No | 1.15 (1.05-1.26) | 0.00233916 |  |
|  | Yes | 1.03 (0.97-1.08) | 0.31397190 |  |
|  | **Hypertension** |  |  | 0.02421514 |
|  | No | 1.21 (1.06-1.37) | 0.00377745 |  |
|  | Yes | 1.04 (0.99-1.09) | 0.11538536 |  |
|  | **Fatty liver** |  |  | 0.10364205 |
|  | No | 1.06 (1.01-1.12) | 0.01513980 |  |
|  | Yes | 1.19 (0.91-1.54) | 0.20006688 |  |
|  | **Glycemic status** |  |  | 1.73e-16 |
|  | Prediabetes | 1.75 (1.55-1.97) | 4.59e-19 |  |
|  | Diabetes | 0.99 (0.93-1.05) | 0.63862879 |  |
|  | **eGFR** |  |  | 0.00188537 |
|  | eGFR <60 | 1.83 (0.89-3.76) | 0.10088269 |  |
|  | eGFR 60-89 | 1.75 (1.39-2.20) | 1.4e-06 |  |
|  | eGFR ≥90 | 1.04 (0.99-1.09) | 0.09231925 |  |
| **AIP** | **Age** |  |  | 0.02234867 |
|  | <60 years | 1.83 (1.67-2.01) | 7.25e-39 |  |
|  | ≥60 years | 1.80 (1.64-1.97) | 2.41e-37 |  |
|  | **Sex** |  |  | 9.06e-16 |
|  | Male | 1.59 (1.46-1.74) | 5.97e-27 |  |
|  | Female | 2.11 (1.91-2.33) | 2.75e-50 |  |
|  | **BMI** |  |  | 0.32599509 |
|  | BMI <24 kg/m2 | 1.57 (1.37-1.81) | 1.34e-10 |  |
|  | BMI ≥24 kg/m2 | 1.95 (1.82-2.10) | 1.28e-72 |  |
|  | **Smoking** |  |  | 0.54712902 |
|  | No | 1.79 (1.66-1.93) | 2.74e-50 |  |
|  | Yes | 2.08 (1.84-2.34) | 2.33e-33 |  |
|  | **Alcohol** |  |  | 0.77900973 |
|  | No | 1.90 (1.75-2.07) | 1.68e-49 |  |
|  | Yes | 1.83 (1.66-2.02) | 1.01e-33 |  |
|  | **Hypertension** |  |  | 0.68990404 |
|  | No | 1.87 (1.69-2.07) | 5.33e-34 |  |
|  | Yes | 1.87 (1.72-2.03) | 6.29e-49 |  |
|  | **Fatty liver** |  |  | 0.03073356 |
|  | No | 1.88 (1.76-2.02) | 6.57e-73 |  |
|  | Yes | 1.67 (1.39-2.00) | 5e-08 |  |
|  | **Glycemic status** |  |  | 2.31e-15 |
|  | Prediabetes | 2.16 (2.00-2.34) | 6.85e-82 |  |
|  | Diabetes | 1.22 (1.09-1.36) | 0.00063443 |  |
|  | **eGFR** |  |  | 0.11408455 |
|  | eGFR <60 | 1.95 (1.21-3.14) | 0.00591980 |  |
|  | eGFR 60-89 | 1.95 (1.69-2.26) | 2.41e-19 |  |
|  | eGFR ≥90 | 1.81 (1.69-1.95) | 4.97e-59 |  |
| **TyG** | **Age** |  |  | 0.03516317 |
|  | <60 years | 1.67 (1.50-1.87) | 1.21e-20 |  |
|  | ≥60 years | 1.80 (1.61-2.01) | 8.22e-25 |  |
|  | **Sex** |  |  | 8.53e-21 |
|  | Male | 1.50 (1.35-1.65) | 2.29e-15 |  |
|  | Female | 2.39 (2.10-2.73) | 1.77e-38 |  |
|  | **BMI** |  |  | 0.00049010 |
|  | BMI <24 kg/m2 | 2.01 (1.64-2.48) | 3.05e-11 |  |
|  | BMI ≥24 kg/m2 | 1.80 (1.65-1.96) | 1.71e-40 |  |
|  | **Smoking** |  |  | 0.91165712 |
|  | No | 1.79 (1.64-1.97) | 8.47e-35 |  |
|  | Yes | 2.05 (1.77-2.38) | 1.15e-21 |  |
|  | **Alcohol** |  |  | 0.85916532 |
|  | No | 1.85 (1.67-2.05) | 8.5e-32 |  |
|  | Yes | 1.90 (1.68-2.14) | 1.65e-24 |  |
|  | **Hypertension** |  |  | 0.79043736 |
|  | No | 1.96 (1.73-2.22) | 7.16e-26 |  |
|  | Yes | 1.80 (1.63-1.99) | 5.24e-30 |  |
|  | **Fatty liver** |  |  | 0.13090685 |
|  | No | 1.82 (1.68-1.98) | 4.74e-45 |  |
|  | Yes | 2.57 (1.87-3.53) | 6.43e-09 |  |
|  | **Glycemic status** |  |  | 7.64e-33 |
|  | Prediabetes | 3.17 (2.79-3.60) | 7.7e-71 |  |
|  | Diabetes | 1.07 (0.96-1.20) | 0.22214063 |  |
|  | **eGFR** |  |  | 0.40567955 |
|  | eGFR <60 | 1.46 (0.92-2.32) | 0.10513733 |  |
|  | eGFR 60-89 | 1.56 (1.32-1.84) | 1.6e-07 |  |
|  | eGFR ≥90 | 1.83 (1.67-2.01) | 1.21e-39 |  |
| **METS-IR** | **Age** |  |  | 6.77e-07 |
|  | <60 years | 3.06 (2.29-4.09) | 5.02e-14 |  |
|  | ≥60 years | 1.03 (0.98-1.09) | 0.20006984 |  |
|  | **Sex** |  |  | 2.24e-26 |
|  | Male | 1.03 (0.98-1.07) | 0.26215789 |  |
|  | Female | 7.41 (5.32-10.31) | 1.51e-32 |  |
|  | **BMI** |  |  | 1.48e-08 |
|  | BMI <24 kg/m2 | 2.47 (1.67-3.65) | 6.63e-06 |  |
|  | BMI ≥24 kg/m2 | 1.24 (1.10-1.40) | 0.00057817 |  |
|  | **Smoking** |  |  | 0.10364205 |
|  | No | 1.06 (0.96-1.16) | 0.22918311 |  |
|  | Yes | 2.32 (1.66-3.25) | 9.58e-07 |  |
|  | **Alcohol** |  |  | 1.36e-05 |
|  | No | 2.59 (2.00-3.34) | 3.2e-13 |  |
|  | Yes | 1.03 (0.98-1.08) | 0.31583004 |  |
|  | **Hypertension** |  |  | 0.00018360 |
|  | No | 2.59 (1.90-3.54) | 2.45e-09 |  |
|  | Yes | 1.05 (0.98-1.13) | 0.16840568 |  |
|  | **Fatty liver** |  |  | 0.91165712 |
|  | No | 1.13 (1.01-1.27) | 0.02984288 |  |
|  | Yes | 6.66 (3.09-14.35) | 1.32e-06 |  |
|  | **Glycemic status** |  |  | 2.41e-69 |
|  | Prediabetes | 15.74 (11.48-21.58) | 1.22e-65 |  |
|  | Diabetes | 1.01 (0.96-1.06) | 0.83901480 |  |
|  | **eGFR** |  |  | 0.00092768 |
|  | eGFR <60 | 8.04 (1.83-35.30) | 0.00576415 |  |
|  | eGFR 60-89 | 5.72 (3.56-9.19) | 5.22e-13 |  |
|  | eGFR ≥90 | 1.06 (0.98-1.14) | 0.16793897 |  |
| **CHG** | **Age** |  |  | 0.00197268 |
|  | <60 years | 1.35 (1.22-1.50) | 3.53e-09 |  |
|  | ≥60 years | 1.26 (1.15-1.37) | 4e-07 |  |
|  | **Sex** |  |  | 7.57e-25 |
|  | Male | 1.11 (1.02-1.21) | 0.01143510 |  |
|  | Female | 1.81 (1.60-2.04) | 2.49e-22 |  |
|  | **BMI** |  |  | 6.96e-06 |
|  | BMI <24 kg/m2 | 1.46 (1.22-1.75) | 4.24e-05 |  |
|  | BMI ≥24 kg/m2 | 1.33 (1.23-1.42) | 1.85e-14 |  |
|  | **Smoking** |  |  | 0.76169996 |
|  | No | 1.32 (1.22-1.43) | 4.64e-12 |  |
|  | Yes | 1.41 (1.25-1.59) | 3.93e-08 |  |
|  | **Alcohol** |  |  | 0.63085470 |
|  | No | 1.38 (1.26-1.51) | 1.74e-12 |  |
|  | Yes | 1.31 (1.18-1.44) | 1.09e-07 |  |
|  | **Hypertension** |  |  | 0.50214778 |
|  | No | 1.49 (1.33-1.66) | 1.56e-12 |  |
|  | Yes | 1.27 (1.16-1.38) | 3.1e-08 |  |
|  | **Fatty liver** |  |  | 0.00329866 |
|  | No | 1.29 (1.20-1.38) | 4.12e-13 |  |
|  | Yes | 3.05 (2.06-4.50) | 2.36e-08 |  |
|  | **Glycemic status** |  |  | 9.55e-47 |
|  | Prediabetes | 3.31 (2.84-3.85) | 1.36e-53 |  |
|  | Diabetes | 1.00 (0.92-1.08) | 0.95409105 |  |
|  | **eGFR** |  |  | 0.13608025 |
|  | eGFR <60 | 1.58 (1.02-2.43) | 0.03904452 |  |
|  | eGFR 60-89 | 1.33 (1.15-1.55) | 0.00016363 |  |
|  | eGFR ≥90 | 1.26 (1.17-1.35) | 1.52e-09 |  |
